# Schizophrenia Misdiagnosis after Capgras and Cotard Delusions in a Patient with Infantile Cystinosis, Cavum Septi Pellucidi, Cavum Vergae and Cavum Veli Interpositi

**DOI:** 10.3390/bs13020157

**Published:** 2023-02-11

**Authors:** João Gama Marques

**Affiliations:** 1 Consulta de Esquizofrenia Resistente, Hospital Júlio de Matos, Centro Hospitalar Psiquiátrico de Lisboa, Avenida do Brasil, 53, 1749-002 Lisboa, Portugal; joaogamamarques@gmail.com; 2 Clínica Universitária de Psiquiatria e Psicologia Médica, Faculdade de Medicina, Universidade de Lisboa, Avenida Professor Egas Moniz, 1649-028 Lisboa, Portugal

**Keywords:** secondary schizophrenia, pseudo-schizophrenia, schizophrenia-like psychosis, Capgras, Cotard, cystinosis, cavum septi pellucidi, cavum veli interpositi, cavum vergae

## Abstract

How many patients with psychosis secondary to genetic conditions or congenital brain malformation have been diagnosed with schizophrenia, since its initial conception more than one hundred years ago? A case report of a young man, with antecedents of Capgras and Cotard syndromes, sent to a schizophrenia treatment-resistant outpatient clinic is presented. Instead of true, primary, idiopathic schizophrenia, a diagnosis of secondary schizophrenia (pseudo-schizophrenia or schizophrenia-like psychosis) was made, corresponding to a secondary psychotic syndrome, with hallucinations and delusions due to congenital cavum septi pellucidi, cavum vergae, cavum veli interpositi and progressive brain atrophy due to cystinosis. Extreme caution is recommended when diagnosing schizophrenia in severely psychotic patients independent of their acute or chronic condition. Schizophrenia shall never be forgotten as the great imitated of medicine.

## 1. Introduction

Cavum septi pellucidi and schizophrenia have been correlated for years [1]. Some studies suggest that neurodevelopmental abnormalities in the midline, and associated limbic structures, of the brain could contribute to what some authors call schizophrenia [2]. An elegant systematic review, with meta-analysis, suggested that only the biggest cavum septi pellucidi is associated with psychosis, but these results should be interpreted with caution, as large studies with community-based samples and greater standardization of methods are needed [3]. 

Infantile or nephropathic cystinosis is a rare autosomal recessive lysosomal storage disorder, classically thought of as a childhood disease with bad prognosis. Nowadays, with modern, lifelong, improved treatments, patients are reaching adulthood more easily. However, survival has a price, with patients suffering increasingly more of other less common complications, such as neurologic dysfunction [4] that can still have a profound impact in their life. 

## 2. Case Presentation

A 32-year-old man came to the treatment-resistant schizophrenia outpatient clinic, asking for a second opinion. The patient presented depressive mood, low-self-esteem, anhedonia, adynamia, asthenia, hypersomnia, polyphagia, and bladder and fecal incontinence, with serious compromise of his social life. He admitted, in the past, auditory hallucinations, autoreferential, persecutory and grandiose delusions. In his electronic clinical records, three psychiatric admissions were found over the past ten years. Different doctors described misidentification with Sosia delusion (Capgras syndrome)—with the patient believing his family had been substituted by doppelgangers with sadistic intent—and ruin delusion (Cotard syndrome)—with the patient complaining that he was kept dead by doctors wanting his internal organs for transplant. The patient had previously been on lorazepam, fluoxetine, sodium valproate, olanzapine, risperidone and amisulpride, reaching full remission. He had participated at the day hospital activities with relative success. While not admitted in the hospital, he was living at home with his family, with some support, regarding the therapeutic project. Still, there was contradictory information regarding the diagnosis: borderline personality disorder, bipolar disorder, schizoaffective psychosis, and schizophrenia.

During his early infancy, he had been diagnosed by pediatric nephrology with infantile (nephropathic) cystinosis, after presenting Fancony syndrome and rickets. Genetic testing revealed cystinosin mutation, at the lysosomal cystine transporter (CNTS) gene with homozygotic deletion at chromosome 17p13. Since then, the patient had been living on a rigorous diet, plus chronic treatment with increasing or alternating doses of cysteamine, phosphocysteamine, mercaptamine bitartrate, enalapril, hydroxycholecalciferol, sodium phosphate, sodium citrate, potassium citrate and potassium chloride. During adolescence, he struggled with shyness and introversion, suffering bullying at school. As a young adult he had temporary experiences with nicotine, caffeine, alcohol, cannabinoids and cocaine. Nevertheless, he managed to finish his master’s thesis in anthropology.

Regarding the patient’s familial history, both the living mother and the deceased father were described as having an unspecified personality disorder. One brother and one sister suffered from depression and alcoholism. Two uncles, one aunt and one cousin were diagnosed with unspecified psychosis. Unfortunately, his family support decayed a lot after his father’s death with lung cancer.

**Figure 1:**
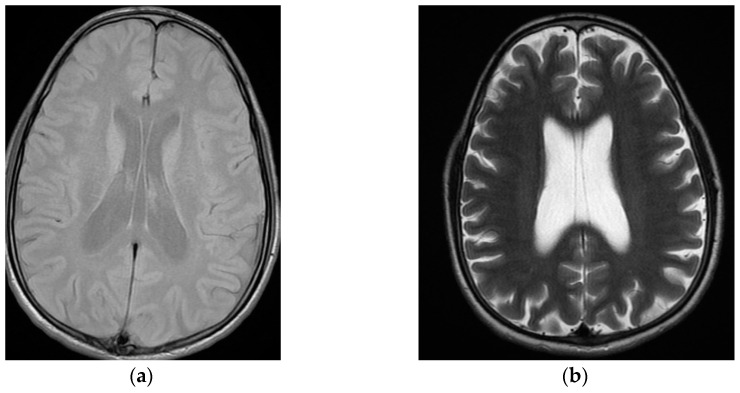
Sequential neuroimaging showing mild progression of diffuse cortical cerebral atrophy with cavum septi pellucidi, cavum vergae, and cavum veli interpositi: (**a**) first brain magnetic resonance imaging (T1), at the index psychiatric admission; (**b**) second brain magnetic resonance imaging (T2), at treatment-resistant schizophrenia outpatient appointment, ten years later.

While at the three past psychiatric admissions, various blood and urine tests, chest radiographs and electrocardiograms were normal. One psychological assessment revealed no signs of personality disorder. One neuropsychological showed a discrepancy between medium verbal and inferior non-verbal intellectual efficiencies, with dyscalculia and deficits in attention and working memory. Two recent electroencephalograms, in a two year span, showed no changes. At the first admission, brain magnetic resonance imaging revealed cavum septi pellucidi and septum vergae (Figure 1a). Ten years later, a new brain magnetic resonance imaging scan confirmed cavum septi pellucidi and septum vergae, also disclosing, for the first time, cavum veli interpositi with mild diffuse encephalic cortical atrophy (Figure 1b).

The patient was diagnosed with secondary psychotic syndrome, with hallucinations and delusions (code 6E61.2), according to the World Health Organization’s International Classification of Diseases, eleventh revision (WHO’s ICD 11), due to cavum septi pellucidi, cavum vergae, cavum veli interpositi and brain atrophy due to cystinosis. A neurosurgery appointment was recommended. Psychoeducation was provided to the patient and his principal caretaker. Group therapy was suggested. Psychiatric medication was adjusted to amisulpride 600 mg and mirtazapine 30 mg, taken together both at night.

## 3. Discussion

For years, anecdotal case reports have described psychosis among patients with cavum septi pellucidi [5], cavum vergae [6], and cavum veli interpositi [7]. More recently, a meta-analysis of hundreds of case reports revealed behavioral disorders in almost 16% of patients with cavum septum pellucidum, cavum vergae and/or cavum veli interpositi [8]. So, an organic psychosis secondary to these brain anomalies is probable, not only possible.

There is no known cause for schizophrenia, so it shall be considered a primary or an idiopathic disorder. This is written in the major psychiatric nosological systems, not only in the WHO’s ICD-11, but also in the American Psychiatric Association’s Diagnostic and Statistical Manual of mental disorders, fifth edition (APA’s DSM-5). Whenever there is a probable cause for psychosis, schizophrenia shall not be diagnosed. Epistemologically speaking, Kraepelin’s dementia praecox concept is obsolete; Bleuler’s schizophrenias idea is outdated, and Schneider’s first rank symptoms are pathognomonic of nothing [9]. On the other hand, even the most bizarre classic psychopathology syndromes, such as the delusion of misidentification in Capgras syndrome [10,11] or the delusion of ruin in Cotard syndrome [12,13], are also not specific of schizophrenia, and should always be the object of a rigorous exclusion of organic cause. For those patients, a psychiatrist should always think of secondary schizophrenia, pseudo-schizophrenia or schizophrenia-like psychosis [14]. Additionally, therefore, in all these cases, the most suitable diagnostic code, the ICD-11’s 6E61, for secondary psychotic syndrome, should be applied instead.

Sometimes, psychiatric patients do not receive the comorbid diagnoses of organic conditions. More often, many patients are sent to psychiatry with the hurried diagnosis of schizophrenia, without the integration of the so-called organic findings. At our personal cohort of two hundred patients previously diagnosed with schizophrenia, we found a new diagnosis of organic psychosis in 25%, with a mean delay, until the correct diagnosis, of twelve years. [15] Among those, there were patients with Huntington chorea [16], Mitsuda psychosis [17], Dandy–Walker syndrome [18], Dalmau autoimmune encephalitis [19], and Lyme disease [20]. We even found, among those unfortunate patients, three cases of psychosis secondary to temporal lobe epilepsy due to cavum septi pellucidi [21].

In this particular patient, the cavum septi pellucidi, the cavum vergae, the cavum veli interpositi, plus the cystinosis-related neurological dysfunction mostly probably had a synergically contribution to the pathophysiology of an organic psychosis, not schizophrenia. Without this kind of rigorous clinical approach, psychiatry will be doomed, keeping on repeating decades of epistemological and nosological mistakes, therefore remaining the underdog of medicine.

## 4. Conclusions

Every case of first-episode psychosis deserves a full medical workup [22,23]. Some authors would even say that every case of last-episode psychosis deserves the same kind of attention [24]. Physicians should ask themselves: why, in the age of neuroimaging, are brain lesions mistaken for psychiatric disorders? [25] Awareness of secondary schizophrenia, pseudo-schizophrenia or schizophrenia-like disorders should be raised to minimize mistakes in diagnosis, theragnosis and prognosis. Caution is recommended when diagnosing schizophrenia in severely psychotic patients independent of their acute or chronic condition. Additionally, last but not least, schizophrenia shall be understood as the great imitated of medicine [26]. 

## Data Availability

Data are unavailable due to privacy restrictions.
